# Molecular and Enzymatic Characterization of Flavonoid 3′-Hydroxylase of *Malus* × *domestica*

**DOI:** 10.3390/plants10091956

**Published:** 2021-09-19

**Authors:** Julia Weissensteiner, Christian Molitor, Silvija Marinovic, Lisa Führer, Syed Waqas Hassan, Olly Sanny Hutabarat, Andreas Spornberger, Karl Stich, Johanna Hausjell, Oliver Spadiut, Christian Haselmair-Gosch, Heidi Halbwirth

**Affiliations:** 1 Institute of Chemical, Environmental and Bioscience Engineering, Technische Universität Wien, Getreidemarkt 9, 1060 Vienna, Austria; julia.weissensteiner@tuwien.ac.at (J.W.); christian.molitor@tuwien.ac.at (C.M.); silvija.marinovic@tuwien.ac.at (S.M.); e1203928@student.tuwien.ac.at (L.F.); olly_hutabarat@yahoo.com (O.S.H.); karl.stich@tuwien.ac.at (K.S.); johanna.hausjell@tuwien.ac.at (J.H.); oliver.spadiut@tuwien.ac.at (O.S.); christian.gosch@tuwien.ac.at (C.H.-G.); 2 Department of Bio-Sciences, University of Wah, Quaid Avenue, Wah 47040, Pakistan; waqas.hassan@uow.edu.pk; 3 Department of Agricultural Engineering, Hasanuddin University, Makassar 90245, Indonesia; 4 Institute of Viticulture and Pomology, University of Natural Resources and Life Sciences, Gregor-Mendel-Straße 33, 1180 Vienna, Austria; andreas.spornberger@boku.ac.at

**Keywords:** *Malus* × *domestica* (apple), flavonoids, dihydrochalcones, chalcones, flavonoid 3-hydroxylation

## Abstract

*Malus* × *domestica* (apple) accumulates particularly high amounts of dihydrochalcones in various tissues, with phloridzin (phloretin 2′-*O*-glucoside) being prevalent, although small amounts of 3-hydroxyphloretin and 3-hydroxyphloridzin are also constitutively present. The latter was shown to correlate with increased disease resistance of transgenic *M.* × *domestica* plants. Two types of enzymes could be involved in 3-hydroxylation of dihydrochalcones: polyphenol oxidases or the flavonoid 3′-hydroxylase (F3′H), which catalyzes B-ring hydroxylation of flavonoids. We isolated two F3′H cDNA clones from apple leaves and tested recombinant *Malus* F3′Hs for their substrate specificity. From the two isolated cDNA clones, only *F3′HII* encoded a functionally active enzyme. In the F3′HI sequence, we identified two putatively relevant amino acids that were exchanged in comparison to that of a previously published F3′HI. Site directed mutagenesis, which exchanged an isoleucine into methionine in position 211 restored the functional activity, which is probably because it is located in an area involved in interaction with the substrate. In contrast to high activity with various flavonoid substrates, the recombinant enzymes did not accept phloretin under assay conditions, making an involvement in the dihydrochalcone biosynthesis unlikely.

## 1. Introduction

**Figure 1:**
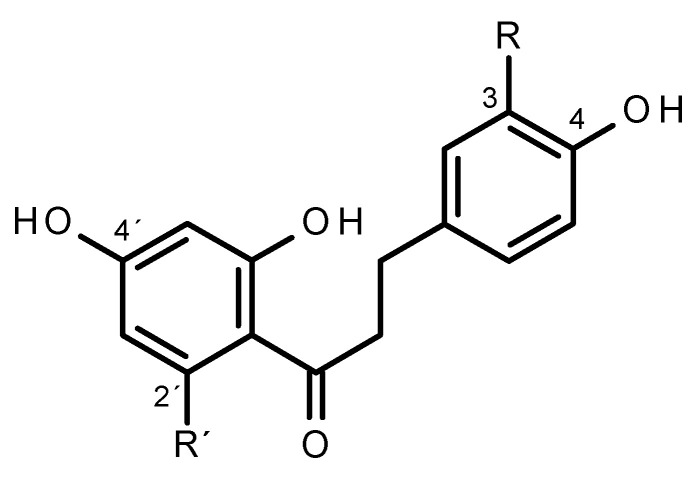
Chemical structures of selected dihydroxychalcones found in Malus species. R=H, R’=OH: Phloretin. R, R’=OH: 3-Hydroxyphloretin. R=H, R’=Glc: Phloretin 2′-*O-*glucoside (phloridzin). R=OH, R’=Glc: 3-Hydroxyphloretin 2′-*O*-glucoside (3-hydroxyphloridzin).

*Malus* × *domestica* (apple) accumulates particularly high amounts of dihydrochalcones in leaves, fruits, and barks, with phloridzin (phloretin 2′-*O*-glucoside) (Figure 1) being the prevalent compound [1]. Formation of dihydrochalcones occur as a side branch of the flavonoid pathway, with *p*-coumaroyl-CoA as common intermediate [2]. The side branch is opened by a *p*-dihydrocoumaroyl-CoA dehydrogenase, which catalyzes the reduction of the double bond of *p*-coumaroyl-CoA [3]. The encoding gene was not unambiguously identified so far, although several candidate genes were suggested [4,5]. *p*-Dihydrocoumaroyl-CoA is further converted by the common chalcone synthase (CHS) to the dihydrochalcone phloretin [3,6], and subsequently glucosylated to provide phloridzin [7,8,9,10]. The special case of apple, with its exceptionally high amount of accumulated dihydrochalcones [11], gave rise to speculation about the physiological function of dihydrochalcones in this species. A role in pathogen defense was controversially discussed [1,12,13,14]. For systematic investigation, sufficiently comprehensive knowledge of the underlying pathway is still lacking.

3-Hydroxyphloridzin, carrying an additional hydroxyl group at position 3 in the B-ring (Figure 1), is also present constitutively, though in very low amounts [1]. Using transgenic *M.* × *domestica* plants, we previously showed that an increase in 3-hydroxylated dihydrochalcones correlates with a lower susceptibility for fire blight and apple scab [15]. 3-Hydroxyphloridzin and 3-hydroxyphloretin can occur as intermediates in the oxidation of phloridzin and phloretin by polyphenol oxidases (PPO). Recently, three recombinant PPOs from *M.* × *domestica* were characterized with tyramine and dopamine as substrates and classified as tyrosinases [16,17]. The suggested involvement of PPOS [18,19] is particularly questionable as in intact cells, PPOs and dihydrochalcones, as with polpyhenols in general, are thought to be kept in separate cell compartments to avoid undirected cell damage by interaction with quinones, which are the final products of polyphenol oxidation by PPOs [20,21].

A second enzyme that could be involved in the B-ring hydroxylation of dihydrochalcones is the cytochrome P450 dependent monooxygenase flavonoid 3′-hydroxylase (F3′H), which catalyzes the introduction of an additional hydroxyl group in the B-ring of various flavonoid classes. F3′H ultimately determines the B-ring-hydroxylation pattern of anthocyanidins, and thereby the color hue of anthocyanin containing tissues. Hydroxylation occurs, however, earlier in the pathway, typically at the flavanone or dihydroflavonol level. In addition, flavonols are common substrates for all F3′Hs [22]. Hydroxylation of anthocyanidins was not observed so far, and leucoanthocyanidins and flavones were shown to be substrates for some, but not all, F3′Hs [23]. Although the hydroxylation of chalcones in the B-ring shows structural similarity to the F3′H reaction, special chalcone 3-hydroxylase (CH3H) enzymes are required, which apparently evolved from F3′Hs [24,25]. Dihydrochalcones are structurally related to chalcones, and we previously showed that they are accepted as substrates by both CH3H and F3′H of the ornamental plant *Cosmos sulphureus*, albeit to a low extent [15]. Recent years saw increasing interest in the metabolic engineering of the dihydrochalcone pathway in various microorganisms to specifically produce dihydrochalcones for nutritional or pharmaceutical purposes [26,27,28]. Thus, the enzymes and genes involved in the dihydrochalcone pathway of apples are also of biotechnological interest. In this frame, a screening of 6 F3′Hs from 6 plants and 1 CH3H was performed to test their suitability for metabolic engineering of 3-hydroxyphloretin formation. Out of those, only the CH3H enzyme was convincing, and two apple F3′Hs were reported to be ‘apparently inactive with phloretin’ [27].

For a better understanding of the flavonoid 3′-hydroxylation, we investigated the substrate specificities of two recombinant *Malus* F3′H for phloretin. Coincidentally, we identified an amino acid essential for the functional activity of F3′H.

## 2. Results

### 2.1. Cloning and Characterization of F3′H

Based on the sequence information available in the NCBI database (FJ919631, FJ919633), full size primers were designed for the isolation of cDNA clones of the two *F3′H* loci found in *Malus* × *domestica*, *MdF3′HI* and *MdF3′HII* (allelic variant *MdF3′HIIb*) [29]. Using mRNA preparations from apple leaves, two cDNA clones were obtained (NCBI accession numbers MH468788 (clone *MdF3′HI*) and MH468789 (clone *MdF3′HII*), each showing an open reading frame of 511 amino acids. In comparison to that of the NCBI sequence FJ919631, the newly isolated *MdF3′HI* cDNA clone showed an amino acid identity of 99.6%, with two nucleotide exchanges resulting in an exchange of amino acids 211 and 224 ( Figure S1a). In comparison to that of the NCBI sequence FJ919633, the newly isolated *MdF3′HII* cDNA showed an amino acid sequence identity of 99.6%, with 6 nucleotide exchanges resulting in an exchange of two amino acids in positions 73 and 457 (Figure S1b). The newly isolated *MdF3′HI* and *MdF3′HII* sequences showed an amino acid identity of 94.4% (Figure S1c).

**Figure 2:**
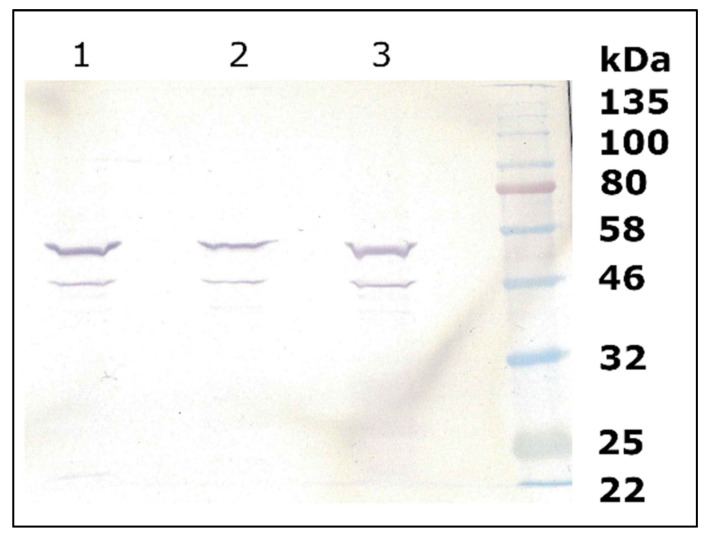
Western blot of recombinant enzyme preparations obtained after heterologous expression in *Saccharomyces cerevisiae*. Picture was doctored to enhance visibility; original is available under Figure S3. Lane 1: MdF3′HI, Lane 2: MdF3′HII, Lane 3: MdF3′HI I22M/S224P. Western blot analysis clearly demonstrated presence of recombinant proteins. Protein band at around 58 kDa shows intact MdF3′H enzyme. MdF3′H seems smaller than calculated size because composition of microsome preparation might have an influence on migration of protein. Band at around 46 kDa is probably a C-terminal digested part of F3′H.

After heterologous expression in yeast, the recombinant proteins were tested for functional activity. Whereas MdF3′HIIb was functionally active, catalyzing the introduction of a hydroxyl group in position 3′ of different flavonoid substrates, repeated attempts to obtain functionally active MdF3′HI were not successful despite both cDNA clones showing a comparable suitability for heterologous expression without codon usage optimization for heterologous expression in yeast (Table S2). In addition, the presence of recombinant protein could be clearly demonstrated by Western blot analysis (Figure 2).

The deduced amino acid sequences of *MdF3′HI* and *MdF3′HII* did not identify amino acid exchanges in the 6 regions previously indicated to be involved in substrate recognition (substrate recognition sites SRS1-6) [30], with the exception of amino acid 211, which was an isoleucine in MdF3′HI instead of the methionine in MdF3′HII and which is located at SRS2 (Figure S1c) Interestingly, this was also one of the amino acid exchanges in our MdF3′HI in comparison with that of MdF3′HI already available in the database (FJ919631) (Figure S1a). 

We therefore performed site-directed mutagenesis of the *MdF3′HI* cDNA clone to test the relevance of the two sites for the functional activity of F3′H. Three recombinant MdF3′HI mutants were produced, MdF3′HI I211M, MdF3′HI S224P, and MdF3′HI I211M S224P. The exchange of serine against proline in position 224 did not influence the activity of recombinant enzyme. After exchange of 211 isoleucine against methionine by site-directed mutagenesis of the cDNA clone, we obtained, however, a functionally active enzyme. Simultaneous exchange of both sites did not seem to have a synergistic effect.

### 2.2. Substrate Specificity of Recombinant Malus F3′Hs

The *MdF3′HI* I211M and *MdF3′HII* cDNA clones were heterologously expressed and the kinetic values of the resulting recombinant enzymes for a broad spectrum of substrates were determined at optimized conditions (Table S3). Naringenin, dihydrokaempferol, and kaempferol were tested as standard substrates for F3′H. In addition, phloretin (dihydrochalcone), isoliquiritigenin (chalcone), apigenin (flavone), and 5-deoxyleucopelargonidin (flavan 3,4-diol) were tested. Incubation of naringenin, dihydrokaempferol and kaempferol in the presence of NADPH led to the formation of the 3′4′-hydroxylated counterparts eriodictyol, dihydroquercetin, and quercetin, respectively. No product formation was observed in the absence of NADPH or when heat-inactivated enzyme preparations were used in the assays. In contrast, apigenin, 5-deoxyleucopelargonidin and isoliquiritigenin were not accepted as substrates. The kinetic data of both enzymes were determined with naringenin, dihydrokaempferol and kaempferol as substrates (Table 1). The values obtained for the substrates were in a similar range. Both recombinant enzymes, MdF3′HI I211M and MdF3′HII, showed highest specificity for the flavonol kaempferol. 

**Figure 3:**
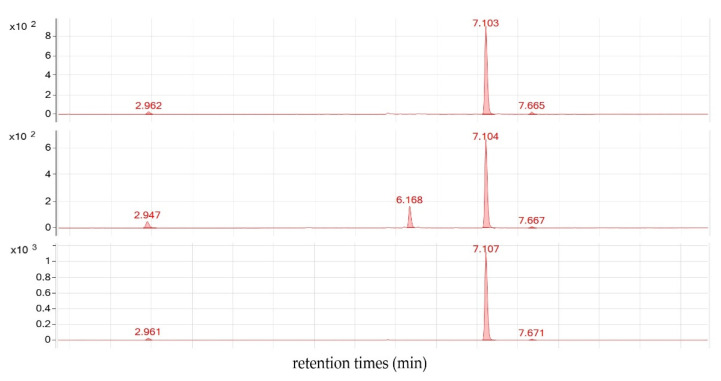
DAD-chromatogram at 280 nm of LC-MS analysis after incubation of phloretin and NADPH in presence of recombinant MdF3′HII (MH468789) (top); CsCH3H; (FJ216429) (center), and CrCYPred (X69791) (bottom).

Most notably, phloretin conversion into the corresponding 3-hydroxyphloretin was not detected with the photo diode array detector in the presence of any of the recombinant *Malus* F3′Hs, even if the incubation time was extended up to 60 min and the amount of recombinant enzyme was increased up to 40 µL (Figure 3). There were, however, small amounts of 3-hydroxyphloretin detected by the more sensitive mass detector, but these were apparently formed by an unspecific enzyme from *S. cerevisiae* because a negative control yeast expressing cytochrome P450 reductase of *Catharanthus roseus* (*CrCypr*) produced similar amounts of 3-hydroxphloretin (Figure S2, Table S4). In contrast, recombinant chalcone 3-hydroxylase from *Cosmos sulphureus*, which was previously shown to accept dihydrochalcones to a moderate extent [15], produced sufficient amounts of 3-hydroxyphloretin to be detected by the DAD (Figure 3).

## 3. Discussion

### 3.1. Hydroxylation of Chalcones in Position 3

The hydroxylation pattern of flavonoids is of physiological relevance, as biological activity is frequently related to the presence of vicinal hydroxyl groups at positions 3′ and 4′ [31]. In line with this, the previously observed decreased pathogen susceptibility of gm-apple trees overexpressing a chalcone 3-hydroxylase [15] could be based on the constitutive higher levels of 3-hydroxyphloridzin present.

In the flavonoid pathway, the hydroxylation pattern of the B-ring is primarily determined by cytochrome P450 dependent monooxygenases, F3′H and flavonoid 3′5′-hydroxylase (F3′5′H). In general, F3′Hs show a broad substrate specificity, catalyzing the hydroxylation of flavanones, dihydroflavonols, and flavonols, as well as in some cases leucoanthocyanidins or flavones, as substrates [23]. Hydroxylation of the closely related chalcones, in contrast, seems to require a particular architecture of the active site and is, therefore, restricted to the closely related CH3Hs [24,25]. Dihydrochalcones clearly show structural relation to chalcones, as both are lacking the heterocyclic C ring of flavonoids. They share, on the other hand, the saturated bond between C2 and C3 with flavanones and dihydroflavonols, which cannot be found in chalcone substrates. Recombinant CH3H and F3′H from *Cosmos sulphureus* converted the dihydrochalcone phloretin to some extent [15], although it is not a natural substrate in this ornamental plant. As dihydrochalcones are the most common soluble polyphenols in *Malus* sp., and 3-hydroxydihydrochalcones are constitutively formed, it could be assumed that F3′H from *Malus* is particularly adapted to the B-hydroxylation of dihydrochalcones. If that had been the case, this would have made the F3′H from *Malus* particularly interesting for metabolic engineering of microbial strains for the specific production of particular dihydrochalcones, which is of increasing interest [27,32]. However, it seems in contrast that phloretin is apparently better accepted by F3′Hs from other plants than by the *Malus* F3′H [15,27]. 

Three sequences encoding F3′H, designated *MdF3′HI*, *MdF3′HIIa,* and *MdF3′HIIb,* were previously isolated from *Malus* tissues (NCBI FJ919631, FJ919632, FJ919633). We can actually distinguish two types of F3′Hs, because *MdF3′HIIa* and *MdF3′HIIb* are only allelic variants, differing only in a few amino acids. Both F3′H types, which show 91% nucleotide sequence identity, were shown to be functionally active by transgenic expression in *Arabidopsis* and tobacco. Screening of the genome sequence of the domesticated apple did not reveal further F3′H candidates.

We isolated two cDNA clones (NCBI accession numbers MH468788 (*MdF3′HI*) and MH468789 (*MdF3′HII*)) from apple leaves, which represent the two different *MdF3′H* types. Each of the clones had two exchanges in the deduced amino acid sequence compared to that of the cDNA clones from the literature. The recombinant MdF3′HII was functionally active, and converted flavanone, dihydroflavonol, and flavonol substrates, but not the flavone, chalcone, or leucoanthocyanidin substrates. Phloretin conversion into 3-hydroxyphloretin was not observed and could only be observed with sensitive MS detection, and it seems to be caused by *S. cerevisiae* enzymes present in the microsomal preparation. The isolated *MdF3′HI* cDNA clone did not encode a functionally active enzyme unless the activity was restored by site-directed mutagenesis of the cDNA clone, leading to an exchange of an amino acid (see Section 3.2). The functionally active MdF3′HI confirmed that phloretin is not a substrate, or at least very weak substrate, for the F3′Hs in *Malus.* The acceptance of leucoanthocyanidins was for stability reasons tested with 5-deoxyleucopelargonidin [23]. As previously reported for F3′H of *Fragaria* (strawberry), F3′H of *Malus* did not convert 5-deoxyleucopelargonidin. Thus, the substrate specificity of the closely related F3′Hs from the two rosaceous species contrasts with the F3′Hs of *Arabidopsis thaliana* and *Tagetes erecta*, from the Brassicaceae and Asteraceae family, respectively [23].

### 3.2. A Methionine in Position 211 Is Essential for Functional Activity

An unexpected side-result of our work was the coincidental identification of an amino acid in the F3′H sequence, which is essential for functionality. The newly isolated cDNA clone *MdF3′HI* showed six nucleotide exchanges in comparison to that of FJ919631 and could not be heterologously expressed into a functionally active enzyme. This could not be explained by technical reasons which may typically occur if a plant gene is heterologously overexpressed in microbes, such as unfavorable codon usage or the occurrence of insoluble protein. As FJ919631 was demonstrated to be functionally active in planta [29], it could be therefore assumed that the two amino acid exchanges could be of relevance. Located at position 211 and 224, they are in proximity to each other and to regions previously suggested to be involved in substrate binding of cytochrome P450-dependent monooxygenases [30]. Isoleucine 211 is part of the substrate binding region 2 (SRS2) and was therefore the more promising candidate for being the key amino acid responsible for the functional inactivity than the serine in position 224, which is a highly conserved proline in the functionally active MdF3′HI and located between SRS2 and SRS3. The exchange of isoleucine 211 into methionine completely restored the functional activity of our MdF3′HI, whereas an exchange of the serine into the proline had no effect. As no crystal structure of F3′H or a sufficiently closely related cytochrome P450-dependent monooxygenase is available, the exact role of methionine 211 for functional activity remains unclear.

## 4. Materials and Methods

### 4.1. Chemicals

(2-^14^C)-Malonyl-coenzyme A (55 mCi/mmol) was obtained from Amersham International (Amersham, UK). [^14^C]-labelled substrates were synthesized as described previously [33] using recombinant enzyme preparations. 3-Hydroxyphloretin was purchased from Apin Chemicals (Oxon, UK), Bovine Serum Albumin, phloretin and phloridzin from Sigma-Aldrich (St. Louis, MI, USA).

BCIP/NBT Color Development Substrate was purchased from Promega (Madison, WI, USA) and Strep-Tactin conjugated to alkaline phosphatase from IBA Lifesciences (Göttingen, Germany).

### 4.2. Plant Material

Young leaves of *M*. × *domestica* cv. Rebella were collected in the experimental orchards of the Institute of Fruit Breeding (JKI, Dresden Pillnitz, Germany) and the Institute of Viticulture and Pomology (University of Natural Resources and Life Sciences, Jedlersdorf, Austria) in spring 2003 and 2004. Plant material was shock-frozen in liquid nitrogen and kept at −80 °C until use.

### 4.3. Cloning and Heterologous Expression of F3′H

Poly(A) tailed RNA from *M.* × *domestica* cv. Rebella was isolated using the µMACS mRNA Isolation Kit (Miltenyi Biotec, Bergisch Gladbach, Germany). Reverse transcription was performed with the SuperScript II Reverse Transcriptase (Invitrogen, Waltham, MA, USA) and the oligo(-dT) anchor primer GACCACGCGTATCGATGTCGAC(T)_16_V. Cloning primers (Table S1) were derived from NCBI database sequences FJ919631 (for *MdF3′H-I*) and FJ919633 (for *MdF3′H-II*) by using the StarPrimer D’Signer software (Version 3.0.0.3, IBA Lifesciences, Göttingen, Germany). PR-PCR was performed with *Pfu* DNA Polymerase (Thermo Scientific, Waltham, MA, USA) and the primer combinations MdF3′HI-SF and MdF3′HI-SR (for *MdF3′H-I*) and MdF3′HIIb-SF and MdF3′HIIb-SR (for *MdF3′H-II*). StarGate^®^ cloning and expression system (IBA Lifesciences, Göttingen, Germany) was used according to the manufacturer’s instructions (protocol version PR26-0023) with *E. coli* strain TOP10 (IBA Lifesciences, Göttingen, Germany) for donor and destination vector generation, and *Saccharomyces cerevisiae strain* INV*Sc*1 (Invitrogen, Waltham, MA, USA) for heterologous expression. In brief, PR-PCR products were inserted into pENTRY-IBA to generate the donor vector. The insert of the donor vector was further subcloned in acceptor vector pYSG-IBA-103 for heterologous expression in *S. cerevisiae*, which allows the heterologous expression of the respective cDNAs as fusion proteins with a C-terminal Twin-Strep-Tag^®^ (tandem peptide WSHPQFEK with an internal linker region). Sequence verification was done by Sanger sequencing (Microsynth Austria AG, Vienna, Austria). Heterologous expression and protein isolation was done as described [15] but additionally CuSO_4_ was added to a final concentration of 0.1 M for induction. Microsomal preparations were used in enzyme assays.

### 4.4. Codon Usage Analysis

Codon usage analysis was performed using the free internet tool available at the GenScript^®^ website (https://www.genscript.com/tools/rare-codon-analysis, accessed on 15 March 2021).

### 4.5. Site-Directed Mutagenesis

Mutants were generated from IBA103 vector constructs using the Q5 Site-Directed Mutagenesis Kit (NewEngland Biolabs, Vienna, Austria). Primers (Table S1) were designed using the NEBaseChanger TMv 1.2.3 provided at http://nebasechanger.neb.com, (accessed on 15 March 2021 and 18 August 2021). The integrity of the constructs was confirmed by commercial sequencing (Microsynth Austria AG, Vienna, Austria).

### 4.6. Western Blot

For analysis of the membrane bound proteins, SDS-PAGE and Western blots were performed. At first, a microsomal preparation was carried out as described before [15]. The samples were directly mixed 1:6 with 6x concentrated Laemmli buffer [34] and heated up on 95 °C for 5 min. After that, the samples were loaded on 12% Polyacrylamide gel. Color Prestained Protein Standard, Broad Range (NEB) was used as a standard. The Mini-Protean Tetra Cell of Bio-Rad was used. The gels were run in SDS-buffer (0.025 M Tris, 0.192 M Glycin, 0.1% SDS, pH 8.3) at 40 mA during the collecting gel and at 80 mA during separating gel. The gel was blotted on PVDF membrane (Trans-Blot Turbo^TM^ Transfer Pack, BioRad Laboratories, Hercules, US) with the Trans-Blot Turbo Transfer System (BioRad Laboratories, Hercules, CA, USA). After blotting, the membrane was incubated in blocking buffer (2% (*w*/*v*) Bovine Serum Albumin, PBS buffer (1.8 mM KH_2_PO_4_, 10 mM Na_2_HPO_4_ × 7 H_2_O, 2.7 mM KCl, 136 mM NaCl, pH 7.4)) at 4 °C overnight. On the next day, the blot was washed three times with binding buffer (0.25% (*v*/*v*) Tween-20, PBS) for 10 min and incubated with the antibody solution (Strep-Tactin conjugated to alkaline phosphatase in PBS buffer) for 2 h. After incubation the blot was washed three times with binding buffer. The blot was stained with the BCIP/NBT Color Development Substrate in alkaline phosphatase buffer (100 mM Tris, 100 mM NaCl, 5 mM MgCl_2_ × 6 H_2_O, pH 9.5).

### 4.7. Enzyme Assays

Protein determination was performed by a modified Lowry procedure with crystalline BSA as the standard [35]. Enzyme assays with recombinant MdF3′HI and MdF3′HII were performed as described recently [3,25] using optimized assay conditions for both enzymes (Table S3) In a final volume of 100 µL, the F3′H standard enzyme assay contained 0.036 nmol [^14^C]-substrate (dihydrokaempferol, kaempferol, naringenin, or phloretin,) 1.5–3 µL recombinant enzyme preparation, 5 µL NADPH (0.83 mg/mL H_2_O), and 55 µL 0.1 M Tris/HCl (pH 6.5–6.75, 0.4% Na-ascorbate *w*/*v*). The reaction mixture was incubated for 10 min at 25 °C. Thereafter, the reaction was stopped by mixing with 70 µL ethyl acetate and 10 µL 100% acetic acid. After centrifugation for 5 min at 10,000× *g* for phase separation, the organic phase was transferred to a precoated cellulose plate (Merck, Darmstadt, Germany) and substrate and products were separated by thin-layer chromatography (TLC) in chloroform/acetic acid/H_2_O (10:9:1, *v*/*v*/*v*). The conversion rates were determined with a TLC linear analyzer (Berthold, Bad Wildbad, Germany). The optimized reaction conditions are summarized in Table S3. For the determination of potential phloretin hydroxylation, the amount of recombinant enzyme preparation was increased up to 40 µL and incubation time up to 60 min.

For LC-MS analysis, three recombinant enzymes were tested: MdF3′HII (*Malus* x *domestica* flavonoid 3′-hydroxylase (MH468789)), CsCH3H (chalcone 3-hydroxylase of *Cosmos sulfureus* (FJ216429) and CrCPR (NADPH-cytochrome P450 reductase from *Catharanthus roseus* (X69791)). The reaction mixtures contained in a final volume of 100 µL: 40 µL *Saccharomyces cerevisiae* INVSc1 microsomal preparation of recombinant produced enzyme, 1.55 mM NADPH, 10 µM substrate in 100 mM HEPES (4-(2-hydroxyethyl)-1-piperazineethanesulfonic acid), pH 7.5. The mixture was incubated for 30 min at 30 °C and the reaction was stopped with 20 µL of 80% acetonitrile/20% acetic acid. After centrifugation at 16,000× *g* for 5 min, the reaction solution was filtered through a 0.22 µM PTFE membrane.

### 4.8. LC-MS Analysis

UPLC was performed on an Agilent 1290 Infinity II System (Agilent, Santa Clara, CA, USA), equipped with a 1290 Infinity Binary Pump (Agilent, product number G7120A), a 1260 Infinity II Diode Array Detector HS (Agilent, product number G7117C), a 1290 Infinity II Multisampler (Agilent, product number G7167G), and a 1290 1290 Infinity II Multicolumn Thermostat (Agilent, product number G7116B).

One µL of extract was injected onto a ZORBAX Eclipse Plus C18 Rapid Resolution column (Agilent, Santa Clara, USA), with a length of 150 mm, an internal diameter of 2.1 mm and a particle size of 1.8 µm at a column temperature of 35 °C and a flow rate of 0.3 mL/min. Eluent A was Millipore™ H_2_O and eluent B was acetonitrile, both with 0.1% formic acid. Solvent gradient was as follows (values in Time [min]): %B: 0.00: 15%; 0.50: 15%; 8.50: 60%; 10.50: 98%; 15.50: 98%; 15.75: 15%; 19.00: 15%, (Post Run Time: 6 min for Equilibration).

After separation, dihydrochalcones were detected by the Agilent 1260 Infinity II Diode Array Detector HS at 287 nm with a bandwidth of 4 nm. Scanning range was 190–600 nm. Identification was performed using an Agilent High-Resolution-y MS 6545 Q-TOF with Electrospray Ion Source Dual AJS ESI, both supplied by the company Agilent (Santa Clara, CA, USA). The main instrumental conditions were as follows: negative ionization mode, MS scan range was from *m*/*z* 100 to 1,000, product ion scan range from *m*/*z* 50 to 350, capillary voltage 3.5 kV for; gas temperature 350 °C; gas flow 10L/min, nebulizer 40 psi, sheath gas temperature 350 °C, sheath gas flow 12 L/min, fragmentor 180 V; skimmer 75 V. Nitrogen was used as nebulizer and auxiliary gas. Data acquisition was carried out using the Agilent Mass Hunter Workstation Data Acquisition (AB Sciex, Foster City, CA, USA) and evaluated using Agilent MassHunter Qualitative Analysis 10.0. Identifications were based on chromatographic elution time, Accurate Mass, MS/MS fragmentation pattern, and comparisons with available standards.

### 4.9. Kinetic Studies

Experiments for determination of kinetic parameters of the recombinant enzymes were performed by varying the substrate concentrations from 0.12 µM to 2.5 µM at a fixed concentration of 0.5 mM NADPH. The amounts of crude microsomal preparations used of MdF3′HI was 5 µg for naringenin, 3 µg for DHK and 1.5 µg for kaempferol and of MdF3′HII 3 µg for naringenin, 2 µg for DHK and 1.5 µg for kaempferol. Data analysis was carried out by nonlinear regression mean values, and standard deviations were calculated based on three repetitions. Calculations and graphs were carried out employing the program OriginPro 2018 (OriginLab).

## 5. Conclusions

Our studies showed that F3′H from apple have a relatively narrow substrate specificity, as they accept, under in vitro conditions, only the most common substrate classes, flavanones, dihydroflavonols, and flavonols. This also confirms that F3′H from apple is not a suitable candidate for metabolic engineering of the dihydrochalcone pathway in microbial strains. On the other hand, the recent case of the gm-orange petunias clearly demonstrated that even if a compound is such a weak substrate, in vitro conversion at standard conditions is not observed, but accumulation of its converted metabolites can be nonetheless observed in a suitable physiological background [36].

In the case of F3′H, a competition with the glucosyltransferases for phloretin as a substrate would be decisive. A high conversion of phloretin into phloridzin, as becomes obvious from the fact that the majority of dihydrochalcones are present in apple in glucosylated form, corresponds well with the very low amounts of 3-hydroxyphloridzin present in apple, and the fact that usually the concentration of free phloretin is higher than that of 3-hydroxyphloridzin [1] could reflect the low substrate specificity of F3′H for phloretin. A final clarification will only be achieved by silencing of the *Malus* F3′Hs and testing the effect on the levels of constitutively present 3-hydroxyphloridzin.

## Figures and Tables

**Table 1:** Kinetic data for recombinant F3′Hs of *Malus.*

MdF3′HI I211M	Naringenin	Dihydrokaempferol	Kaempferol
*K*_m_-value [µM]	2.11 ± 1.5	0.42 ± 0.06	0.21 ± 0.07
*V*_max_ [µM/s]	0.73 ± 0.37	0.47 ± 0.04	0.35 ± 0.12
*k_cat_* [1/s]	0.00036	0.00038	0.0013
*k_cat_*/*K*_m_ [µM^−1^ s^−1^]	171	905	6190
*Md*F3′HII			
*K*_m_-value [µM]	0.31 ± 0.10	0.31 ± 0.03	0.20 ± 0.02
*V*_max_ [µM/s]	0.50 ± 0.20	0.33 ± 0.01	0.33 ± 0.02
*k_cat_* [1/s]	0.0003	0.0003	0.0004
*k_cat_*/*K*_m_ [µM^−1^ s^−1^]	1000	1000	2000

## Data Availability

All data supporting the findings are contained in the manuscript and its Supplementary Files.

## Supplementary Materials

The following are available online at https://www.mdpi.com/article/10.3390/plants10091956/s1, Figure S1: Pairwise alignments of the deduced amino acid sequences of F3′H cDNA clones of *Malus × domestica*, Figure S2: Secondary mass spectra of 3-hydroxyphlotin obtained during LC-MS analysis, Figure S3: Original of Figure 2. Western blot of the recombinant enzyme preparations obtained after heterologous expression in *Saccharomyces cerevisiae*, Table S1: Primers used for cloning cDNAs from *M. × domestica*, Table S2: Comparison of key values obtained from the codon usage analysis in the two *MdF3′H* cDNA clones, Table S3: Optimized standard assay conditions for the recombinant F3′Hs of *Malus*, Table S4: Confirmation of 3-hydroxyphloretin formation from phloretin in the presence of NADPH and enzyme preparations after heterologous expression in yeast by LC–MS.
