# Supplementary material for: Molecular and Enzymatic Characterization of Flavonoid 3′-Hydroxylase of Malus × domestica
Source: Plants (Basel). 2021 Sep 19;10(9):1956. doi: 10.3390/plants10091956 (PMC8469728; doi:10.3390/plants10091956)
Supplement: Supplementary file 1 [file plants-10-01956-s001.zip › plants-1386852-supplementary.pdf]

Supplementary Material

Supplementary Figure S1. Pairwise alignments of the deduced amino acid sequences of *F3′H* cDNA clones of *Malus* × *domestica.* Comparison of a: newly isolated MdF3'HI (MH468788) with NCBI FJ919631 (Han et al. 2010), b: newly isolated MdF3'HII (MH468789) with NCBI FJ919633 (Han et al. 2010), and c: the newly isolated MdF3'HI (MH468788) *and* MdF3'HII (MH468789). Differences in the amino acid sequences are highlighted in grey-shading. Regions serving as substrate recognition sites (SRS1-6) according to Gotoh (1992) are underlined.

a

```
                  1                                                      50
F3'HI_FJ919631    MFVLIVFTVV FAFFLYRIFA PGGSRHSLPL PPGPKPWPVV GNLPHLGPVP
        MdF3'HI   MFVLIVFTVV FAFFLYRIFA PGGSRHSLPL PPGPKPWPVV GNLPHLGPVP

                  51                                                    100
F3'HI_FJ919631    HHSLAALARQ YGPLMHLRLG FVDVVVAASA SVASQFLKTH DANFSSRPPN
        MdF3'HI   HHSLAALARQ YGPLMHLRLG FVDVVVAASA SVASQFLKTH DANFSSRPPN

                  101   SRS1                                            150
F3'HI_FJ919631    SGAKHLAYNY QDLVFAPYGP RWRLLRKISS VHLFSGKALD DLKHVRQEEV
        MdF3'HI   SGAKHLAYNY QDLVFAPYGP RWRLLRKISS VHLFSGKALD DLKHVRQEEV

                  151                                                   200
F3'HI_FJ919631    GVLAHGLASA GSKPVNLAQL LNVCTVNALG RVMVGRRLFG NGMGGEDPKA
        MdF3'HI   GVLAHGLASA GSKPVNLAQL LNVCTVNALG RVMVGRRLFG NGMGGEDPKA

                  201   SRS2                                 SRS3       250
F3'HI_FJ919631    DEFKSMVVEM MVLAGVFNIG DFIPSLEWLD LQGVAGKMKK LHKRFDAFLT
        MdF3'HI   DEFKSMVVEM IVLAGVFNIG DFISSLEWLD LQGVAGKMKK LHKRFDAFLT

                  251                                           SRS4    300
F3'HI_FJ919631    AIVEEHKRSR GGKHVDMLTT LLSLKEDADG EGAKLTDTEI KALLLNMFTA
        MdF3'HI   AIVEEHKRSR GGKHVDMLTT LLSLKEDADG EGAKLTDTEI KALLLNMFTA

                  301                                                   350
F3'HI_FJ919631    GTDTSSSTVE WAIAELLRHP KILAQLQQEL DQVVGRDRLV TESDLPNLTY
        MdF3'HI   GTDTSSSTVE WAIAELLRHP KILAQLQQEL DQVVGRDRLV TESDLPNLTY

                  351             SRS5                                  400
F3'HI_FJ919631    LQAVIKETFR LHPSTPLSLP RMATESCEIN GFHIPKGATL LVNVWAVSRD
        MdF3'HI   LQAVIKETFR LHPSTPLSLP RMATESCEIN GFHIPKGATL LVNVWAVSRD

                  401                                                   450
F3'HI_FJ919631    PDQWSEPLEF RPERFMSGGE KPNVDIRGND FEVIPFGAGR RICAGMSLGL
        MdF3'HI   PDQWSEPLEF RPERFMSGGE KPNVDIRGND FEVIPFGAGR RICAGMSLGL

                  451                                   SRS6            500
F3'HI_FJ919631    RMVSLMTATL VHGFDWTLAD GLTPEKLNMD EAYGLTLQRA APLMVHPRNR
        MdF3'HI   RMVSLMTATL VHGFDWTLAD GLTPEKLNMD EAYGLTLQRA APLMVHPRNR

                  501      511
F3'HI_FJ919631    LAPHAYNASS S
        MdF3'HI   LAPHAYNASS S
```

b

```
                    1                                                 50
F3'HIIb_FJ919633   MFVLIFFTVV LAFFLYRLFA PGGSRHALPL PPGPKPWPVV GNLPHLGPVP
        MdF3'HII   MFVLIFFTVV LAFFLYRLFA PGGSRHALPL PPGPKPWPVV GNLPHLGPVP

                    51                                                100
F3'HIIb_FJ919633   HHSLAALARQ YGPLMHLRLG FVDVVVAASA SVASQFLKTH DANFSSRPPN
        MdF3'HII   HHSLAALARQ YGPLMHLRLG FADVVVAASA SVASQFLKTH DANFSSRPPN

                    101    SRS1                                       150
F3'HIIb_FJ919633   SGAKHLAYNY QDLVFAPYGP RWRMLRKISS VHLFSGKALD DLKHVRQEEV
        MdF3'HII   SGAKHLAYNY QDLVFAPYGP RWRMLRKISS VHLFSGKALD DLKHVRQEEV

                    151                                               200
F3'HIIb_FJ919633   GVLAHGLASA GSKPVSLGQL LNVCTVNALG RVMVGRRLFG DGGGREDQKA
        MdF3'HII   GVLAHGLASA GSKPVSLGQL LNVCTVNALG RVMVGRRLFG DGGGREDQKA

                    201   SRS2                                SRS3    250
F3'HIIb_FJ919633   DEFKSMVVEM MVLAGVFNIG DFIPALEWLD LQGVAGKMKK LHKRFDAFLT
        MdF3'HII   DEFKSMVVEM MVLAGVFNIG DFIPALEWLD LQGVAGKMKK LHKRFDAFLT

                    251                                           SRS4 300
F3'HIIb_FJ919633   AIVEDHKRSG EGKHVDMLTT LLSLTDDADG EGAKLTDTEI KALLLNMFTA
        MdF3'HII   AIVEDHKRSG EGKHVDMLTT LLSLTDDADG EGAKLTDTEI KALLLNMFTA

                    301                                               350
F3'HIIb_FJ919633   GTDTSSSTVE WAIAELLRHP KILAQLQQEL DQVVGRDRLV TESDLPNLTY
        MdF3'HII   GTDTSSSTVE WAIAELLRHP KILAQLQQEL DQVVGRDRLV TESDLPNLTY

                    351               SRS5                            400
F3'HIIb_FJ919633   LQAVIKETFR LHPSTPLSLP RMASESCEIN GFHIPKGATL LVNVWAISRD
        MdF3'HII   LQAVIKETFR LHPSTPLSLP RMASESCEIN GFHIPKGATL LVNVWAISRD

                    401                                               450
F3'HIIb_FJ919633   PAQWSEPLEF RPERFLPGGE KPNVDVKGND FEVIPFGAGR RICAGMTLGL
        MdF3'HII   PAQWSEPLEF RPERFLPGGE KPNVDVKGND FEVIPFGAGR RICAGMTLGL

                    451                                  SRS6         500
F3'HIIb_FJ919633   RMVSLMIATL VHGFDWTLAD GLTPEKLNMD EAYGLTLQRA APLMVHPRNR
        MdF3'HII   RMVSLMTATL VHGFDWTLAD GLTPEKLNMD EAYGLTLQRA APLMVHPRNR

                    501     511
F3'HIIb_FJ919633   LAPHAYNASS P
        MdF3'HII   LAPHAYNASS P
```

c

```
             1                                                 50
  MdF3'HI    MFVLIVFTVV FAFFLYRIFA PGGSRHSLPL PPGPKPWPVV GNLPHLGPVP
 MdF3'HII    MFVLIFFTVV LAFFLYRLFA PGGSRHALPL PPGPKPWPVV GNLPHLGPVP

             51                                               100
  MdF3'HI    HHSLAALARQ YGPLMHLRLG FVDVVVAASA SVASQFLKTH DANFSSRPPN
 MdF3'HII    HHSLAALARQ YGPLMHLRLG FADVVVAASA SVASQFLKTH DANFSSRPPN

             101 SRS1                                             150
  MdF3'HI    SGAKHLAYNY QDLVFAPYGP RWRLLRKISS VHLFSGKALD DLKHVRQEEV
 MdF3'HII    SGAKHLAYNY QDLVFAPYGP RWRMLRKISS VHLFSGKALD DLKHVRQEEV

             151                                              200
  MdF3'HI    GVLAHGLASA GSKPVNLAQL LNVCTVNALG RVMVGRRLFG NGMGGEDPKA
 MdF3'HII    GVLAHGLASA GSKPVSLGQL LNVCTVNALG RVMVGRRLFG DGGGREDQKA

             201  SRS2                                   SRS3  250
  MdF3'HI    DEFKSMVVEM IVLAGVFNIG DFISSLEWLD LQGVAGKMKK LHKRFDAFLT
 MdF3HII     DEFKSMVVEM MVLAGVFNIG DFIPALEWLD LQGVAGKMKK LHKRFDAFLT

             251                                         SRS4  300
  MdF3'HI    AIVEEHKRSR GGKHVDMLTT LLSLKEDADG EGAKLTDTEI KALLLNMFTA
 MdF3'HII    AIVEDHKRSG EGKHVDMLTT LLSLTDDADG EGAKLTDTEI KALLLNMFTA

             301                                              350
  MdF3'HI    GTDTSSSTVE WAIAELLRHP KILAQLQQEL DQVVGRDRLV TESDLPNLTY
 MdF3'HII    GTDTSSSTVE WAIAELLRHP KILAQLQQEL DQVVGRDRLV TESDLPNLTY

             351           SRS5                                400
  MdF3'HI    LQAVIKETFR LHPSTPLSLP RMATESCEIN GFHIPKGATL LVNVWAVSRD
 MdF3'HII    LQAVIKETFR LHPSTPLSLP RMASESCEIN GFHIPKGATL LVNVWAISRD

             401                                              450
  MdF3'HI    PDQWSEPLEF RPERFMSGGE KPNVDIRGND FEVIPFGAGR RICAGMSLGL
 MdF3'HII    PAQWSEPLEF RPERFLPGGE KPNVDVKGND FEVIPFGAGR RICAGMTLGL

             451                              SRS6            500
  MdF3'HI    RMVSLMTATL VHGFDWTLAD GLTPEKLNMD EAYGLTLQRA APLMVHPRNR
 MdF3'HII    RMVSLMTATL VHGFDWTLAD GLTPEKLNMD EAYGLTLQRA APLMVHPRNR

             501        511
  MdF3'HI    LAPHAYNASS S
 MdF3'HII    LAPHAYNASS P
```

Supplementary Figure S2. Secondary mass spectra of 3-hydroxyphlotin obtained during LC-MS analysis after incubation of phloretin and NADPH in the presence of recombinant MdF3'HII (MH468789) (top) and CrCYPred (X69791) (bottom). Additional data available in Suppl. Table S4.

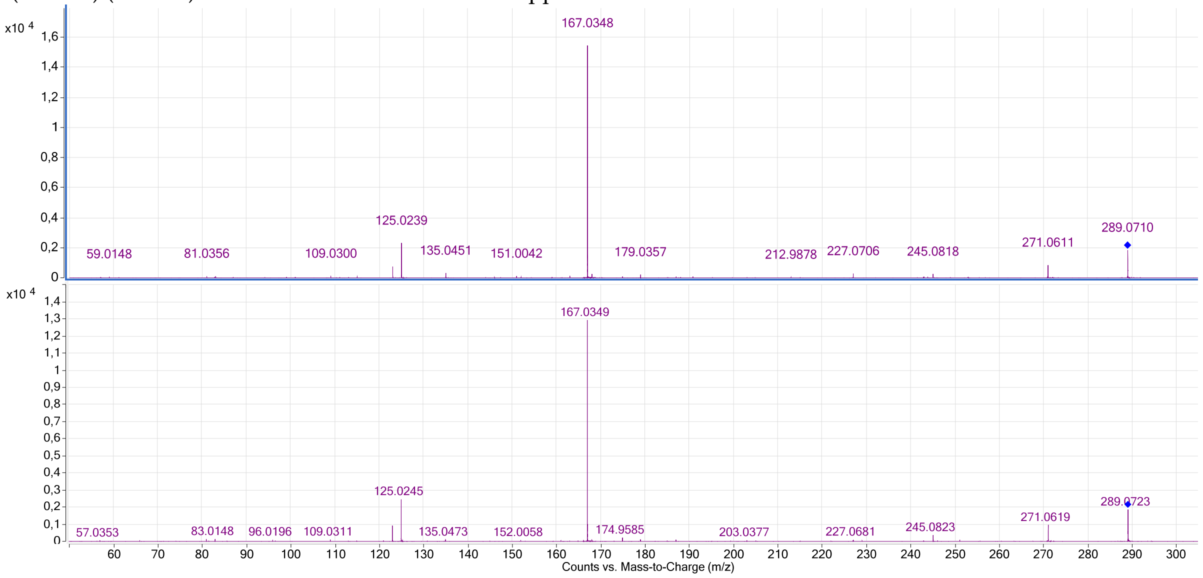

Supplementary Figure S3. Original of figure 2. Western blot of the recombinant enzyme preparations obtained after heterologous expression in *Saccharomyces cerevisiae*.

Lane 1: MdF3′H HI, Lane 2: MdF3′H HII, Lane 3: MdF3′H HI I22M/S224P. The Western blot analysis clearly demonstrated the presence of the recombinant proteins. The protein band at around 58 kDa shows the intact MdF3′H enzyme. MdF3′H seems smaller than the calculated size because the composition of the microsome preparation might have an influence of the migration of the protein. The band at around 46 kDa is probably a C-terminal digested part of the F3′H.

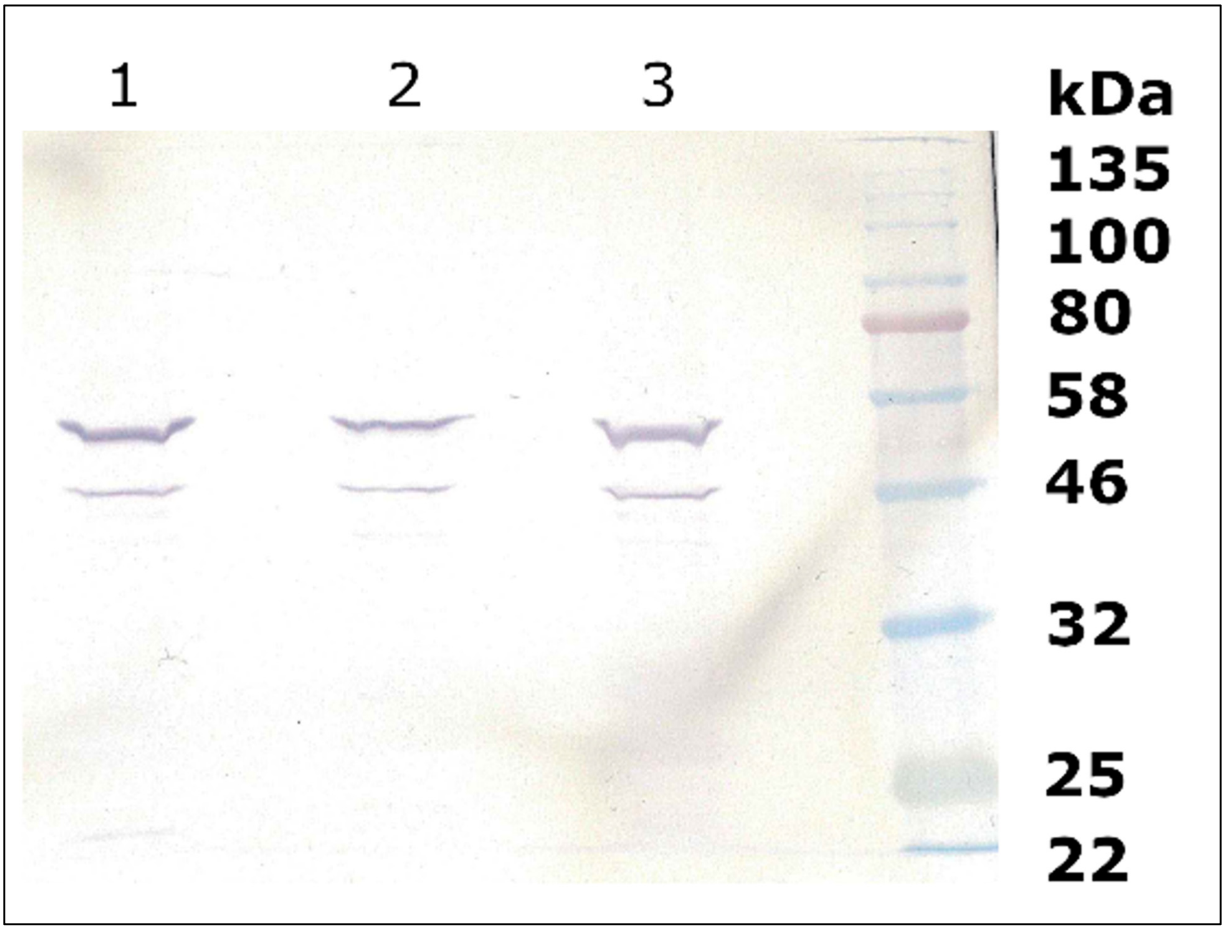

Supplementary Table S1. Primers used for cloning cDNAs from *M.* × *domestica*. Start codon ATG highlighted in bold. StarGate combinatorial sites *AATG* and *TCCC* highlighted in black. StarCombinase1 recognition area highlighted in grey. The asterisk refers to a phosphorothioate internucleotide linkage to protect primers from nuclease degradation. T_a: PCR annealing temperature.

**Clone**	**Primer (5'-3')**		**T_a (°C)**
MdF3´HI	MdF3´HI-SF	AGCGGCTCTTCA**ATG**TTTGTTCTCATAGTCTTCACC*G	58
	MdF3´HI-SR	AGCGGCTCTTCTCCCAGATGATGATGCATTGTATGC*A	
MdF3´HII	MdF3´HIIb-SF	AGCGGCTCTTCA**ATG**TTTGTTCTCATATTCTTCACC*G	58
	MdF3´HIIb-SR	AGCGGCTCTTCTCCCAGGTGATGACGCATTATATG*C	
MdF3´HI I211M/ S224P	F_MdF3´HI211M/ S224P	AACATCGGCGACTTCATCCCCTCCCTAGAGTGGCTGGAC	65
	R_MdF3´HI211M/ S224P	GAATACTCCGGCCAACACCATCATCTCCACCACCATGGAC	
MdF3´HI I211M	F_MdF3H HI I211M	GGTGGAGATGATGGTGTTGGCCG	65
	R_MdF3H HI I211M	ACCATGGACTTGAACTCATCC	
MdF3´HI S224P	F_MdF3'H_S224P	CGACTTCATCCCCTCCCTAGAGT	61
	R_MdF3'H_S224P	CCGATGTTGAATACTCCG	

Supplementary Table S2. Comparison of key values obtained from the codon usage analysis in the two *MdF3'H* cDNA clones. Analysis was performed by the free online-tool https://www.genscript.com/tools/rare-codon-analysis.

cDNA clone	*MdF3'HI* (MH468788)	*MdF3'HIIb* (MH468789)
**Codon Adaptation Index**	0.56	0.56
**GC content [%]**	57.24	57.60
**Codon Frequency Distribution [%]**	13	12
**Negative repeat elements**	0	0

Supplementary Table S3. Optimized standard assay conditions for the recombinant F3'Hs of *Malus*

MdF3'HII	**Naringenin**	**Dihydrokaempferol**	**Kaempferol**
**pH optimum**	6.75	6.5	6.75
**Temperature optimum [°C]**	25	n.d.	n.d.
**Time linearity [min]**	10	10	10
**Protein linearity [µg]**	3	2	1.5
***MdF3'HI* I211M**			
**pH optimum**	6.75	6.75	6.75
**Temperature optimum [°C]**	25	n.d.	n.d.
**Time linearity [min]**	15	15	15
**Protein linearity [µg]**	5	3	0.6

*n.d.: not determined

Supplementary Table S4. Confirmation of 3-hydroxyphloretin formation from phloretin in the presence of NADPH and enzyme preparations after heterologous expression in yeast by LC–MS. *Md*F3'HII: flavonoid F3'-hydroxylaseII of *Malus* x *domestica* (MH468789); *Cs*CH3H; chalcone 3-hydroxylase of *Cosmos sulfureus* (FJ216429); NADPH-cytochrome P450 reductase of *Catharanthus roseus* (X69791).

**Product formed by**	**RT (min)**	**m/z Parent-Ion**	**Daughter-Ions m/z**	**Daughter-Ions Rel. Peak (%)**
*Md*F3'HII	6.25	289.0708	167.0348	100.00
			125.0239	14.66
			271.0611	5.40
			123.0459	4.73
			245.0818	1.59
*Cs*CH3H	6.25	289.0723	167.0348	100.00
			125.0246	23.31
			123.0452	6.05
			271.0608	4.03
			245.0817	2.27
*Cr*Cypred	6.25	289.0714	167.0349	100.00
			125.0245	18.64
			271.0619	7.25
			123.0458	6.88
			245.0823	2.81
3-OH-Phloretin Standard	6.25	289.0719	167.0349	100.00
			125.0246	18.15
			123.0452	7.00
			271.0613	5.36
			245.0809	3.74
